# Impact of butorphanol combined with propofol general anesthesia on laboratory indicators and reproductive outcomes in elderly patients undergoing oocyte retrieval

**DOI:** 10.1097/MD.0000000000048487

**Published:** 2026-04-24

**Authors:** Jie Rao, Jichuan Wang, Liwen Liu, Yuanming Xu, Juanbao Peng, Yi Mo, Arshad Mehmood, Minghua Shi, Gaosheng Su

**Affiliations:** a Department of Anesthesiology, Guangxi Zhuang Autonomous Region Reproductive Hospital, Nanning, Guangxi, China; b Central Sterile Supply Department, Guangxi Zhuang Autonomous Region Reproductive Hospital, Nanning, Guangxi, China; c Department of Science and Education, Guangxi Zhuang Autonomous Region Reproductive Hospital, Nanning, Guangxi, China; d State Key Laboratory of Digital Medical Engineering, School of Biological Science and Medical Engineering, Southeast University, Nanjing, China; e Department of Reproductive Medicine, Guangxi Zhuang Autonomous Region Reproductive Hospital, Nanning, Guangxi, China.

**Keywords:** butorphanol, elderly, laboratory indicators, oocyte retrieval, propofol, reproductive outcomes

## Abstract

This study investigates the impact of butorphanol combined with propofol general anesthesia on laboratory indicators and reproductive outcomes in elderly patients undergoing oocyte retrieval and explores the rationality and safety of its clinical application. A total of 3418 elderly infertile patients underwent transvaginal oocyte retrieval from November 2020 to October 2022. The patients were divided into a control group (non-anesthesia group, n = 250) and a study group (butorphanol combined with propofol general anesthesia group, n = 3168). The basic information (age and body mass index [BMI] of both partners, fertilization method), laboratory indicators (number of oocytes retrieved, fertilization rate, blastocyst formation rate, usable blastocyst rate), and early pregnancy outcome indicators (clinical pregnancy rate, ectopic pregnancy rate, early miscarriage rate) were compared between the 2 groups. There were no statistically significant differences in the age, BMI, and fertilization methods of the couples between the 2 groups (*P* > .05). The study group had a higher number of oocytes retrieved than the control group, with a statistically significant difference (*P* < .05). However, there were no statistically significant differences in fertilization rate, blastocyst formation rate, and usable blastocyst rate between the 2 groups (*P* > .05). There were no statistically significant differences in clinical pregnancy rate, ectopic pregnancy rate, and early miscarriage rate between the 2 groups (*P* > .05). Butorphanol combined with propofol general anesthesia can increase the number of oocytes retrieved in elderly patients undergoing oocyte retrieval without affecting other laboratory indicators or pregnancy outcomes, and it can be safely applied in clinical practice.

## Graphical abstract

**Figure:**
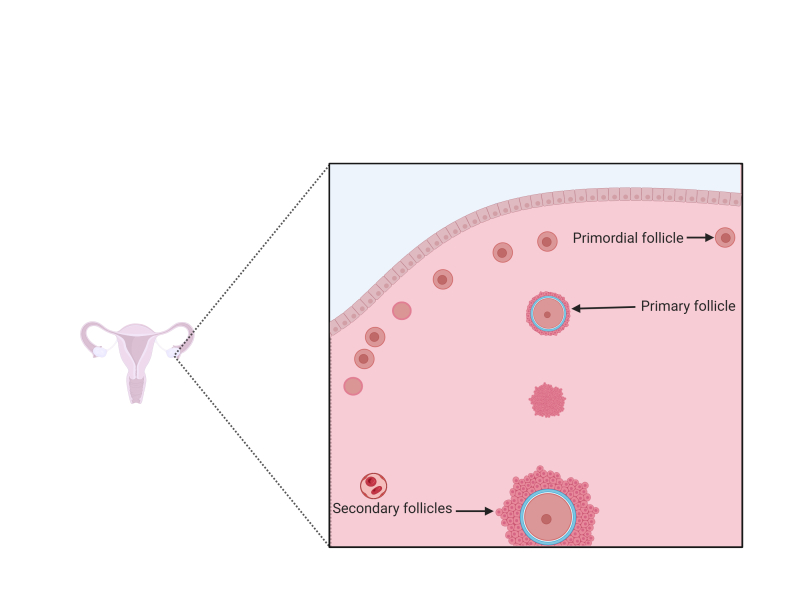

**Key points**

- General anesthesia with butorphanol and propofol significantly increased the number of oocytes retrieved in elderly patients undergoing IVF.
- Fertilization rate, blastocyst formation, and usable blastocyst rate remained unaffected by the anesthetic regimen.
- Clinical pregnancy, ectopic pregnancy, and early miscarriage rates were comparable between anesthetized and non-anesthetized groups.
- Butorphanol offers effective analgesia with a favorable safety profile and minimal adverse effects in elderly patients.
- Combining butorphanol with propofol is a rational and safe approach to improve procedural cooperation and comfort during oocyte retrieval without compromising reproductive outcomes.

## 1. Introduction

With the passage of time, women’s reproductive capacity gradually declines, and increasing age itself is an independent risk factor affecting pregnancy outcomes and fertility.^[1]^ Typically, by the age of 35, women enter the advanced age for reproduction, and at this stage, about 30% of women face the risk of infertility.^[2]^ Older women whose fertility has declined or is nearly lost are more likely to rely on assisted reproductive technologies (ART) to conceive.^[3]^ Elderly infertile patients are more prone to emotional issues, such as depression and anxiety.^[4]^ Some patients worry that the use of anesthetic drugs during the oocyte retrieval process of ART may have adverse effects on pregnancy outcomes.^[5]^ Therefore, they endure severe pain without using any anesthesia or analgesic drugs during the transvaginal oocyte retrieval procedure.^[6]^ However, patients who do not use anesthesia or analgesic methods often cannot tolerate pain or the adverse stress of tension during oocyte retrieval and cannot fully cooperate with the surgeon to perform the procedure, leading to a decrease in the number of oocytes retrieved and the number of embryos.^[7]^

To reduce the anxiety and other adverse stress of infertile patients and allow them to undergo oocyte retrieval surgery in a pain-free and comfortable condition, this study retrospectively analyzed the data of elderly infertile patients who had undergone transvaginal oocyte retrieval to clarify the safety of butorphanol combined with propofol^[8]^ general anesthesia in transvaginal oocyte retrieval.

## 2. Materials and methods

### 2.1. Study subjects

This study selected 4098 infertile patients aged 35 years and above who underwent in vitro fertilization-embryo transfer (IVF-ET) and transvaginal oocyte retrieval from November 2020 to October 2022 at Guangxi Zhuang Autonomous Region Reproductive Hospital. After excluding 680 patients based on the established inclusion and exclusion criteria, a total of 3418 eligible patients were included as study cases, and their medical records were retrospectively reviewed. The patients were divided into a control group (non-anesthesia group, n = 250) and a study group (butorphanol combined with propofol general anesthesia group, n = 3168).

*Inclusion criteria:* Patients undergoing transvaginal oocyte retrieval for IVF-ET treatment who were aged ≥35 years. Patients who received butorphanol combined with propofol intravenous general anesthesia or no anesthesia at all. Patients who had signed an informed consent form. Patients whose fertilization method was either conventional in IVF or intracytoplasmic sperm microinjection (ICSI).

*Exclusion criteria:* Patients who underwent oocyte retrieval under regional anesthesia or conscious sedation analgesia. Patients who received other general anesthesia methods. Patients with incomplete clinical data. The experiments were performed at the Reproductive Hospital of Guangxi Zhuang Autonomous Region and were approved by the Experimental Ethics Committee of the Reproductive Hospital of Guangxi Zhuang Autonomous Region, China. All methods were carried out in accordance with relevant guidelines and regulations. All methods are reported in accordance with the Helsinki guidelines.

### 2.2. Grouping

Patients were divided into 2 groups based on whether anesthesia was administered during the transvaginal oocyte retrieval procedure. Control group (non-anesthesia group): This group consisted of patients who voluntarily requested no anesthesia or analgesic methods for the transvaginal oocyte retrieval procedure. Before the surgery, the surgical process was explained to the patients to alleviate their panic. Patients were instructed to empty their bladder and maintain the lithotomy position. Intravenous access was established for fluid administration. After entering the operating room, patients received routine mask oxygenation (4 L/min), and their blood pressure, heart rate, respiration, and pulse oximetry saturation were monitored. Study group (butorphanol combined with propofol general anesthesia group): This group included patients who requested general anesthesia for the transvaginal oocyte retrieval procedure. All patients were fasted for more than 6 hours and restricted from drinking for more than 4 hours before the surgery. Before the start of the surgery, patients were intravenously administered butorphanol (Jiangsu Hengrui Medicine Co., Ltd., National Medicine Standard H20020454) at a dose of 0.01 mg/kg and propofol (Guangdong Jiabo Pharmaceutical Co., Ltd., National Medicine Standard H20051842) at a dose of 1.5 to 2.0 mg/kg. Depending on the duration of the surgery or the need for abdominal compression, additional doses of propofol (0.5 mg/kg per administration) were given as needed. Other procedures were the same as in the control group.

### 2.3. Observation indicators

The basic information of the 2 groups of patients was compared, including age, body mass index (BMI), and fertilization methods. The laboratory data of the 2 groups of patients were analyzed and compared, including the number of oocytes retrieved, fertilization rate, blastocyst formation rate, and usable blastocyst rate. The differences in pregnancy outcomes between the 2 groups were further observed, including clinical pregnancy rate, ectopic pregnancy rate, and early (within 12 weeks) miscarriage rate.

### 2.4. Statistical analysis

Data were analyzed using SPSS version 26.0 (IBM Corp.). For normally distributed continuous data, the *t* test was used, and results were expressed as mean ± standard deviation. Categorical data were analyzed using the chi-square (χ^2^) test and presented as percentages (frequency/total number of cases) [% (n/N)]. A *P*-value <.05 was considered statistically significant.

## 3. Results

### 3.1. Comparison of basic information

The comparison between the 2 groups of patients showed no statistically significant differences in the age, BMI, and fertilization methods of both partners (*P* > .05). The specific results are shown (Table 1).

**Table 1:** Comparison of basic information between the 2 groups.

Group	Case	Female age (yr)	Male age (yr)	Female BMI (kg/m^2^)	Male BMI (kg/m^2^)	Fertilization method IVF/(IVF + ICSI)
Study group	3168	37.6 ± 1.6	38.9 ± 4.6	22.9 ± 3.1	24.6 ± 3.6	81.3% (2576/3168)
Control group	250	37.7 ± 1.6	39.0 ± 4.0	22.5 ± 2.8	24.7 ± 3.2	78.8% (197/250)
χ^2^/*t*		−1.434	−0.506	1.947	−0.482	0.956
*P*		.152	.613	.052	.630	.328

BMI = body mass index, ICSI = intracytoplasmic sperm microinjection, IVF = in vitro fertilization.

### 3.2. Comparison of laboratory indicators between the 2 groups

The comparison of the number of oocytes retrieved between the 2 groups showed a statistically significant difference (*P* < .05). Patients who received general anesthesia with butorphanol and propofol had a significantly higher number of oocytes retrieved (9.1 ± 4.3) compared to those who did not receive anesthesia. However, there were no statistically significant differences in fertilization rate, blastocyst formation rate, and usable blastocyst rate between the 2 groups (*P* > .05). The specific results are shown (Table 2).

**Table 2:** Comparison of laboratory indicators between the 2 groups.

Indicator	Control group (n = 3168)	Study group (n = 250)	χ^2^/*t*	*P*
Number of oocytes retrieved	9.1 ± 4.3	4.3 ± 3.7	−4.654	.000
Fertilization rate	71.1 (20,722/29,145)	72.3 (777/1075)	0.702	.402
Blastocyst formation rate	60.5 (8477/14,011)	60.0 (251/419)	0.061	.805
Blastocyst rate can be utilized	60.8 (5154/8477)	61.0 (153/251)	0.009	.923

### 3.3. Comparison of reproductive outcomes

The comparison of clinical pregnancy rate, ectopic pregnancy rate, and early miscarriage rate between the 2 groups of patients showed no statistically significant differences (*P* > .05). The specific results are shown (Table 3).

**Table 3:** Comparison of reproductive outcomes between the 2 groups.

Indicator	Control group (n = 3168)	Study group (n = 250)	χ^2^	*P*
Clinical pregnancy rate (%)	39.3 (716/1821)	39.7 (54/136)	0.008	.929
Ectopic pregnancy rate (%)	0.8 (6/716)	1.9 (1/54)	0.000	.989
Early miscarriage rate (%)	11.2 (80/716)	9.3 (5/54)	0.187	.665

## 4. Discussion

With the implementation of the 2-child policy and the increasing number of women who marry and give birth later in life, the number of elderly infertile patients has been increasing year by year, and they often need to use ART technology to conceive.^[9]^ Transvaginal oocyte retrieval is an important part of the IVF-ET process.^[10]^ During this procedure, when the puncture needle passes through tissues, such as the vagina and ovaries, or when the abdomen is compressed to adjust the position of the ovaries, patients can experience strong discomfort and pain. Anesthesia and analgesia are needed to eliminate this strong adverse stress.^[11]^ The success rate of elderly infertile women in becoming pregnant and giving birth to healthy babies is significantly reduced, the possibility of spontaneous abortion is greatly increased, and the risks of pregnancy complications, neonatal congenital defects,^[12]^ and adverse emotions, such as depression and anxiety in patients, are also increased.^[13]^ Some patients worry that the drugs used for anesthesia and analgesia during oocyte retrieval may have adverse effects on laboratory indicators and pregnancy outcomes, so they choose not to use any anesthesia or analgesia.^[14]^ However, patients who do not use anesthesia or analgesia often cannot fully cooperate with the oocyte retrieval procedure due to pain and tension, resulting in a decrease in the number of oocytes retrieved and the number of embryos available for transfer.^[15]^ Elderly patients have a significant decline in ovarian reserve and fewer mature follicles, so the number of oocytes retrieved during ART is relatively low. A decrease in the number of oocytes retrieved also significantly increases the risk of having no embryos available for transfer.^[16]^ In addition, patients who do not use anesthesia or analgesia may also experience adverse events, such as increased blood pressure and arrhythmia due to pain and tension, reducing patient comfort and satisfaction and causing significant psychological stress.^[17]^ Therefore, it is important to study and explore a safe oocyte retrieval anesthesia method that has no adverse effects on pregnancy outcomes to reduce the anxiety of infertile patients and allow them to undergo oocyte retrieval surgery in a safe, painless, and comfortable environment.

Currently, anesthesia and analgesia methods used for transvaginal oocyte retrieval include regional anesthesia, conscious sedation analgesia, and general anesthesia. General anesthesia has the advantages of rapid onset, definite analgesic effect, quick recovery, simple operation, and flexible drug combination. It allows patients to fully cooperate with the surgical procedure and provides good surgical conditions for the surgeon, which helps to increase the number of oocytes retrieved.^[18]^ Therefore, it is the most commonly used method to relieve pain during transvaginal oocyte retrieval. Propofol, with its rapid action, easy control, and lack of significant accumulation, has become the most widely used general anesthetic. However, due to its weak analgesic effect, the dose of propofol needs to be increased when used alone to achieve the desired anesthetic effect. However, data show that high concentrations of propofol in follicular fluid when using large doses of propofol may affect oocyte development.^[19]^ Therefore, in clinical practice, intravenous anesthesia methods combining propofol with opioid receptor agonists or opioid receptor agonist–antagonists are commonly used for transvaginal oocyte retrieval to reduce the dose of propofol while ensuring appropriate anesthetic depth.

Butorphanol is a drug that has both opioid receptor agonist and antagonist effects. It can rapidly activate κ-opioid receptors and partially block μ-opioid receptors without affecting σ-opioid receptors. The drug indirectly inhibits cyclooxygenase, preventing the formation of prostaglandins due to injury and thus blocking the transmission of pain signals within the nervous system to achieve an analgesic effect.^[20]^ Compared to sufentanil, butorphanol is more effective in treating visceral pain.^[21]^ Compared to μ-receptor agonists (sufentanil), butorphanol has fewer side effects, such as reduced risks of respiratory depression, hypotension, bradycardia, nausea, vomiting, itching, and physical dependence, and it provides a degree of postoperative analgesia.^[22]^ Butorphanol used during childbirth has a higher safety profile than other opioid receptor agonists, with minimal adverse effects on the Apgar score of newborns and negligible drug content in breast milk, and it can be used for pain control in postpartum women.^[23]^

Results showed that the number of oocytes retrieved in the study group was significantly higher than that in the control group. Patients who received general anesthesia with butorphanol and propofol could fully cooperate with the puncture and abdominal compression procedures, allowing the surgeon to extract oocytes from the follicular fluid under optimal conditions. In contrast, patients without anesthesia often could not eliminate their anxiety or tolerate the pain and other stimuli and could not cooperate with the oocyte retrieval procedure, which may have led to some oocytes in the follicular fluid not being retrieved. There were no statistically significant differences between the study group and the control group in fertilization rate, blastocyst formation rate, usable blastocyst rate, clinical pregnancy rate, ectopic pregnancy rate, and early miscarriage rate. General anesthesia with butorphanol and propofol does not have adverse effects on oocyte quality, other laboratory indicators, or pregnancy outcomes. Clinical doses of butorphanol^[24]^ provide appropriate sedation and analgesia, reducing the dose of propofol and avoiding potential adverse effects from high doses of propofol. General anesthesia with butorphanol^[25]^ and propofol has minimal impact on the respiratory and circulatory functions of patients, ensuring stable respiration and circulation during the perioperative period and a stable internal environment, which minimizes the impact on follicular fluid and oocytes. It does not affect these laboratory indicators and pregnancy outcomes. Reducing the duration of surgery is an important factor in avoiding the impact of drugs on laboratory indicators and pregnancy outcomes. Patients in the study group could better cooperate with the oocyte retrieval procedure, resulting in shorter surgery times. This avoids high concentrations of drugs in the follicular fluid and reduces the contact time between the drugs and the oocytes, not adversely affecting laboratory indicators and pregnancy outcomes.

## 5. Limitation

This study was a single-center retrospective study, which ensured the uniformity of assisted reproductive technology and anesthesia procedures, but it also had some limitations: The sample size of the comparison group was relatively small. The study only focused on elderly patients, which may introduce bias. The study did not continuously follow-up on later pregnancy outcomes, such as the proportion of live births and the incidence of birth defects.

## 6. Future perspectives

Future research could involve multiple centers to validate findings across diverse patient populations. This approach will increase the sample size and statistical power, providing more reliable conclusions regarding the impact of anesthesia methods on reproductive outcomes in elderly patients undergoing oocyte retrieval. Including patients of various age groups and medical backgrounds to reduce potential biases. This will provide a more comprehensive understanding of how anesthesia methods impact different demographics, ensuring that findings are applicable to a wider range of patients undergoing oocyte retrieval. Implementing long-term follow-up protocols is important to assess pregnancy outcomes, including live birth rates and the incidence of birth defects.^[26]^ Structured follow-up visits and data collection will offer a holistic view of the impact of anesthesia methods on both immediate and long-term reproductive health outcomes. Incorporating patient-reported outcomes and satisfaction measures will provide insights into patient experiences and preferences. Surveys, interviews, and qualitative assessments can help develop patient-centered anesthesia practices, improving overall patient care. Investigating new anesthetic agents and techniques with improved safety profiles and better reproductive outcomes is crucial. This research can lead to the development of optimized anesthesia protocols that minimize risks while maximizing patient comfort and reproductive success.

## 7. Conclusion

General anesthesia with butorphanol and propofol increases the number of oocytes retrieved in elderly patients undergoing oocyte retrieval without affecting other laboratory indicators or pregnancy outcomes, and it can be safely applied in clinical practice.

## Acknowledgments

The authors would like to acknowledge the technical support provided by the Guangxi Zhuang Autonomous Region Reproductive Hospital.

## Author contributions

**Conceptualization:** Jie Rao.

**Data curation:** Jichuan Wang.

**Formal analysis:** Liwen Liu.

**Funding acquisition:** Yi Mo.

**Investigation:** Yuanming Xu.

**Methodology:** Juanbao Peng.

**Project administration:** Minghua Shi.

**Supervision:** Gaosheng Su.

**Writing – original draft:** Arshad Mehmood.

**Writing – review & editing:** Arshad Mehmood.
